# Between-word processing and text-level skills contributing to fluent reading of (non)word lists and text

**DOI:** 10.1007/s11145-024-10533-8

**Published:** 2024-04-03

**Authors:** Sietske van Viersen, Angeliki Altani, Peter F. de Jong, Athanassios Protopapas

**Affiliations:** 1 Department of Development and Education of Youth in Diverse Societies, Utrecht University, Utrecht, The Netherlands; 2 Department of Special Needs Education, University of Oslo, Oslo, Norway; 3 Research Institute of Child Development and Education, University of Amsterdam, Amsterdam, The Netherlands; 4 Department of Counseling, Higher Education, and Special Education, University of Maryland, Maryland, USA

**Keywords:** Fluency, Language skills, Serial naming, Text reading, Word reading, Word recognition

## Abstract

Recent studies have shown that fluent reading of word lists requires additional skills beyond efficient recognition of individual words. This study examined the specific contribution of between-word processing (sequential processing efficiency, indexed by serial digit RAN) and subskills related to text-level processing (vocabulary and syntactic skills) to a wide range of reading fluency tasks, while accounting for within-word processes (i.e., those involved in phonological recoding, orthographic decoding, and sight word reading). The sample included 139 intermediate-level (Grade 3, *n* = 78) and more advanced (Grade 5, *n* = 61) readers of Dutch. Fluency measures included simple and complex lists of words and nonwords, and a complex text. Data were analyzed through hierarchical regressions and commonality analyses. The findings confirm the importance of between-word processing for fluent reading and extend evidence from simple word lists and texts to complex word lists and texts, and simple and complex lists of nonwords. The findings hold for both intermediate-level and more advanced readers and, as expected, the contribution of between-word processing increased with reading-skill level. Effects of vocabulary were generally absent, aside from a small effect on text reading fluency in Grade 3. No effects of syntactic skills were found, even in more advanced readers. The results support the idea that once efficient individual word recognition is in place, further fluency development is driven by more efficient between-word processing. The findings also confirm that vocabulary may be less prominent in processing mechanisms underlying fluent word identification in transparent orthographies, across reading levels.

**Supplementary Information:**

The online version contains supplementary material available at 10.1007/s11145-024-10533-8.

## Introduction

Reading fluency requires the ability to accurately and rapidly read series of words (van Viersen et al., 2022). Many theories suggest that reading fluency is primarily based on the accurate and rapid reading of individual words. That is, they posit that fluent reading is based on within-word processes irrespective of whether these words are presented one by one or as sequences (as in word lists or sentences). However, recent evidence indicates that fluent reading of series of words requires additional skills beyond efficient recognition of individual words (Altani et al., 2017b; Protopapas et al., 2013a). For example, Protopapas et al. (2013a) argued that reading series of words fluently requires, in addition to within-word processes, the cascaded processing of multiple words.

The term “between-word processing” is used to refer to skills involved in the simultaneous processing of multiple words. Their precise nature is currently unknown but may include timing/scheduling, coordination, previewing/preparation, control of parallel operations, shielding from interference, etc. Between-word processing goes beyond within-word processing, which refers to subskills involved in intra-word processing of individual words and their constituent parts (e.g., phonological recoding, orthographic decoding, sight word reading). One aspect of between-word processing that has attracted research attention concerns the efficiency of sequential processing. In particular, sequential processing efficiency concerns the ability to coordinate the processing of multiple successive words at the same time (Altani et al., 2020; Gordon & Hoedemaker, 2016; Protopapas et al., 2013a, 2018); recent studies have argued that sequential processing efficiency may be a missing link in understanding the development from effortful word decoding to fluent word list and text reading (e.g., Altani et al., 2020).

Furthermore, Hudson et al. (2009) describe reading fluency as a complex multi-faceted construct that entails about every subskill of reading, from those associated with lower-level within-word processing to those related to more advanced text-level processing (see also Wolf & Katzir-Cohen, 2001). Yet, much is still unknown about how subskills related to word- and text-level processing interact, especially across commonly used fluency measures and various grade levels. Therefore, in the present study we examined the specific contribution of between-word processing (as indicated by sequential processing efficiency) and subskills related to text-level processing (i.e., vocabulary and syntactic skills) to a wide range of reading fluency tasks, while accounting for within-word processes, in intermediate-level (Grade 3) and more advanced (Grade 5) readers of Dutch.

### Fluent reading of multiple words and individual word recognition

Between-word processing only becomes relevant once processing of individual words is efficient enough to permit the processing of multiple words. For example, eye tracking research has confirmed that both beginning readers and poor readers spend much of their cognitive resources on decoding a single word and have limited capacity to allow adjacent words to be partially processed or retained in working memory at the same time (see e.g., Huestegge et al., 2009; Jones et al., 2013; Yan et al., 2013). Within-word processing concerns all processes that operate on a single word, either holistically or on its (partially) individuated constituents. Thus, all aspects typically investigated in studies of single word reading, including phonological recoding and orthographic decoding as well as direct lexical access (sight word reading). Word-level subskills associated with within-word processing can be measured with a range of discrete tasks (i.e., presenting items individually), including discrete nonword reading and discrete word reading, as well as discrete digit naming as a measure of lexical access speed. Each of these tasks is considered to capture separate but partially overlapping aspects of reading individual words.

Naturally, identification of individual words is most efficient when they can be read by sight; hence when a word and its constituent parts can be recognized as a whole entity at a single glance (Ehri, 2005, 2014). Ehri and Wilce (1983) used measures of response latency to individually presented words or digits (i.e., discrete word reading and discrete digit naming) to illustrate that skilled readers can identify and name individual number words as quickly as they can name single digits.^1^ More recently, Protopapas et al. (2018) reported that discrete word reading and discrete digit naming aligned closely with a “discrete” factor (with loadings of .86 and .90, respectively) across Grades 3 and 5. These kinds of findings have indicated that, once automatized, individual stimuli can be named/read rapidly via a shared process mapping visual-orthographic to phonological forms.

It can seem intuitive that quick and effortless word recognition should largely determine the speed with which a child can read a series of words. Accordingly, individual word recognition speed was long assumed to be the sole factor underlying fluent word-list reading (Ehri, 1997, 2005; Schwanenflugel et al., 2006; Wolf & Katzir-Cohen, 2001). However, processing multiple words in lists (i.e., serial display) is not the same as processing individual words in isolation (i.e., discrete display; see Altani et al., 2020; Protopapas et al., 2018). Recent evidence showed that the prediction of reading fluency of word lists by individual word recognition speed is far from perfect (Altani et al., 2017b; Protopapas et al., 2013a; van Viersen et al., 2022). Moreover, the contribution of individual word recognition and lexical access (as indexed by discrete word reading and discrete digit naming) to fluent reading decreases considerably with development (Altani et al., 2020; de Jong, 2011). This is where between-word processing becomes relevant. It has been hypothesized that the simultaneously available words in word-list fluency tasks introduce additional processing demands at the between-word level. Between-word level processing is thought to entail the processing of successive words simultaneously at different levels, also denoted as *cascaded* processing (Protopapas et al., 2013a, 2018). For example, in a string of four words, the first word can still be articulated while the second word is phonologically mapped, the third word is viewed, and the fourth word is already previewed. Between-word processing thus happens at the same time across successive items.

Sequential processing efficiency is the subskill that has been found to specifically account for processing sequences of words that are simultaneously available in lists or texts. It is generally indexed by a serial digit-naming task, also known as “rapid automatized naming” or simply RAN (Altani et al., 2020; de Jong, 2011; de Jong & van den Boer, 2021; Protopapas et al., 2018). While many studies on RAN have tried to pinpoint what serial digit naming measures (e.g., phonological or orthographic processing, processing speed, remnants of paired associate learning, and/or flexibility; see Kirby et al., 2010; Norton & Wolf, 2012, for overviews), we use this RAN task to capture specific aspects of its nature: Serial digit naming, which is the sequential processing of numbers, is believed to mimic the between-word processing involved in reading a *series* of words in which each *individual* word can be read by sight. Essentially, naming a series of familiar digits allows for whole-item retrieval and efficient symbol-to-sound mapping at the single-item level, placing the major variability on this measure in the efficiency with which adjacent items are processed sequentially (Protopapas et al., 2018).

There is strong evidence for the significance of sequential processing efficiency (indexed by serial digit naming) in the development of fluent reading, beyond processes required in individual item processing (indexed by discrete digit naming), across languages (Altani et al., 2017a, 2018, 2020; van Viersen et al., 2022; de Jong, 2011; Protopapas et al., 2013a; van den Boer et al., 2016; van den Boer & de Jong, 2015). However, previous studies mainly used reading fluency measures of word lists and texts with simple (short, high-frequency) words that are easily read by sight. Therefore, it is crucial to examine whether the importance of sequential processing efficiency is also evident in more complex reading fluency measures, such as word lists and texts with complex (longer, lower-frequency) words that pose greater demands on individual word processing.

### Fluent reading of connected text

Fluent reading of connected text requires additional processing skills besides efficient (within- and between-) word-level processing. Most importantly, a certain extent of semantic and syntactic processing is needed (e.g., Fuchs et al., 2001; Hudson et al., 2009; Ouellette, 2006; van Silfhout et al., 2015). Evidence suggests that both typical and struggling readers can use vocabulary and syntactic knowledge to aid word identification while reading connected text (e.g., Lane et al., 2009; Mokhtari & Thompson, 2006; Nation & Snowling, 1998; Ricketts et al., 2016; West et al., 1983). More specifically, semantic probability, identification of anaphoric referents, and use of connectives have been found to support reading fluency of sentences and texts (Crosson & Lesaux, 2013; Frisson et al., 2005; Perfetti, 1995; van den Bosch et al., 2018).

Regarding the role of subskills related to text-level processing, Kim (2015) showed that vocabulary and syntactic skills are independent predictors of text-reading fluency in Korean beginning readers. In a subsequent study with Korean beginning readers, Kim (2020) found that the effect of vocabulary on text-reading fluency was mediated by word-reading fluency, while the effect of syntactic skills on text-reading fluency ran through listening comprehension and was not significant. Direct effects were not found. Despite mixed outcomes, these studies suggest that vocabulary and syntactic skills are relevant for text-reading fluency and should be considered when investigating the underlying processes involved in more complex fluency tasks.

Although vocabulary is important for the construction of meaning in sentences and has been long associated with text-level comprehension processes, it is also relevant for fluent reading of lists of unrelated words. Most typical readers should be able to identify all words in these tasks, either through decoding of letters or letter clusters, or through instant word recognition. However, being familiar with the meaning of a word may speed up identification when a word cannot be read by sight (e.g., Taylor et al., 2015). Yet, evidence for this supposed mechanism is generally limited to associations between measures of vocabulary and fluent word reading, and findings vary based on orthographic transparency. For example, studies on English-speaking readers have shown that vocabulary aids fluent reading of words irrespective of regularity early in reading development (Ricketts et al., 2016), whereas in advanced readers vocabulary is mainly related to irregular word reading (e.g., Krepel et al., 2021; Nation & Snowling, 2004; Ricketts et al., 2007). Additionally, evidence from French-speaking children showed that vocabulary is an independent and unique predictor of word-list reading fluency after accounting for decoding skills (Ouellette, 2006; see Kim, 2020, for a similar finding in Korean). In comparison, findings from Dutch, which has a more transparent orthography than English and French, suggest that the association between vocabulary and word-list reading fluency is rather weak (de Jong, 2011; de Jong & van der Leij, 2002). As it is unclear what conditions affect associations between vocabulary and reading fluency outcomes, and whether the influence of vocabulary may differ across reading development, it is vital to further assess the role of vocabulary in fluent reading across multiple relevant tasks and reading-skill levels.

### Changes across development

Several studies have shown that the association between individual word recognition and fluent reading of word lists is strong in beginning readers, but steadily decreases with increasing reading skill (e.g., Altani et al., 2020; de Jong, 2011; Protopapas et al., 2013a, 2018). This suggests that fluent word-list reading changes over time and becomes less similar to an individual word recognition task. In contrast, the association between sequential processing efficiency (i.e., serial digit RAN) and word-list reading fluency increases over time (Altani et al., 2018, 2020; de Jong, 2011; Protopapas et al., 2013a). By Grade 5 to 6, sequential processing efficiency predicts word-list fluency better than individual word recognition speed (Protopapas et al., 2018). The underlying construct of word-list fluency thus seems to converge with that of sequential processing efficiency by the end of primary school (Protopapas et al., 2018; van den Boer et al., 2016). The finding that sequential processing efficiency becomes more important for fluency once individual words are increasingly read by sight, supports the claim that between-word processing provides a missing link in understanding the developmental transition from beginners' word-by-word reading to fluent word-list and text reading (Altani et al., 2020). Yet, more research is needed to learn how sequential processing efficiency contributes to measures of reading fluency across development, and to understand the timing of developmental shifts in underlying processes. This approach is also essential for determining when subskills related to text-level processing might come into play and how their contributions may be influenced by processing demands at the word level.

### Measures of reading fluency

To extend previous findings beyond tasks with short and familiar high-frequency words and simple texts, this study also included complex word lists and more demanding texts as reading fluency outcomes. Complex word lists contain unrelated words of increasing length and decreasing frequency, which are commonly used for screening and diagnostic purposes (e.g., Test of Word Reading Efficiency [TOWRE]; Torgesen et al., 2012). De Jong (2011) found similar correlations of reading simple and complex word lists with the recognition of individual words as well as with sequential processing efficiency, as indexed by serial naming. This suggests similar involvement of individual word recognition and sequential processing efficiency in fluent reading of simple and complex word lists. Also, both simple and complex word lists showed a decreasing relation with individual word recognition and an increasing relation with sequential processing efficiency across development. For subskills related to text-level processing, effects of vocabulary may emerge in more opaque orthographies (e.g., Ouellette, 2006; Ricketts et al., 2007) but seem less likely in a relatively transparent orthography such as Dutch (see de Jong & van der Leij, 2002; de Jong, 2011, for weak correlations between complex word-list reading and vocabulary).

For text-reading fluency as an outcome, the influence of vocabulary is well-documented (see e.g., Fuchs et al., 2001; Hudson et al., 2009; Jenkins et al., 2003). However, less is known about the contribution of subskills related to word-level reading fluency. Previous findings are limited to a contribution of sequential processing efficiency to short, syntactically simple texts in Greek readers of all reading levels and intermediate-level and advanced readers of English (Altani et al., 2020), and to fluent reading of a passage in Italian Grade 6 children (Zoccolotti et al., 2014). Combined contributions of word- and text-level skills were found in a previous study (van Viersen et al., 2022) on sentence reading fluency in the same sample of Grade 3 children as the current study. Both sequential processing efficiency and receptive vocabulary contributed to reading fluency of sentences in intermediate-level readers (Grade 3). There was no effect of syntactic skills.

To cover a wider range of reading fluency tasks, we also included simple and complex lists of nonwords in the present study. Reading lists of nonwords is common practice during screening and in clinical settings (e.g., Phonemic Decoding Efficiency subtest of TOWRE; Torgesen et al., 2012). It provides important information about the development of subskills related to word-level processing while excluding effects of vocabulary. Previous studies have suggested that readers employ different processes *within* words when encountering nonwords varying in length—and possibly difficulty—depending on their reading level (e.g., van den Boer & de Jong, 2015; van den Boer et al., 2016). This implies that efficiency in *between*-word processing of nonwords may also change across development, but possibly at a different rate than for real words.

### Current study

In this study, we specifically sought to investigate (a) sequential processing efficiency at various points during development (in Grades 3 and 5), (b) its importance within a range of measures typically used to assess reading fluency, and (c) how skills typically associated with word-level and text-level processes may uniquely contribute to various reading fluency outcomes. Our sample comprised Dutch children learning to read in Dutch, a semi-transparent language with a complex syllable structure (Seymour et al., 2003). Dutch children generally attain a high level of reading accuracy by the end of Grade 2. From that age on, further development of literacy skills focuses on attaining fluency (van Viersen et al., 2018). The children in our study are expected to function on average at a still developing intermediate (Grade 3) and more advanced (Grade 5) level. The current study differs from our previous study (van Viersen et al., 2022) by also including a sample of Grade 5 children, allowing for a developmental perspective, and addition of fluency measures with nonwords and a complex text, enabling a broader perspective on reading fluency.

The first research question (RQ1, see Table 1) focused on the unique and shared contribution of sequential processing efficiency to fluent reading of simple and complex lists of words and nonwords and connected text in intermediate and more advanced readers. The following research questions (RQ 2–4) focused on the contribution of vocabulary and syntactic skills to fluent reading of (non)word lists and connected text across reading-skill levels. The corresponding hypotheses are also listed in Table 1.

**Table 1:** Research questions and hypotheses

Research question	Hypothesis
RQ1: To what extent does sequential processing efficiency as a between-word process contribute to fluent reading across fluency measures after controlling for within-word processes?	H1: Serial digit naming (representing sequential between-item processing efficiency) is expected to contribute uniquely to all fluency measures, with its influence increasing across development
RQ2: To what extent does vocabulary contribute to fluent reading of complex word lists?	H2: Vocabulary is expected to contribute uniquely to fluent reading of complex words lists after word-level processes are taken into account, at least among intermediate-level readers
RQ3: Does vocabulary contribute uniquely to text-reading fluency after accounting for word-level processes?	H3: Vocabulary is expected to contribute uniquely to text-reading fluency across development, after word-level processes are taken into account
RQ4: How and when do syntactic skills come into play during fluent reading of texts?	H4: Syntactic skills are expected to contribute uniquely to text-reading fluency after word-level processes are taken into account, but only in more advanced readers

## Methods

### Participants

Participants were 139 Dutch children from Grades 3 (*n* = 78, 52.6% girls) and 5 (*n* = 61, 57.4% girls). Children were recruited through two large school boards and came from four different schools in the middle and west of the Netherlands. Parents signed written informed consent for their child’s participation and children gave oral assent before testing. Data collection was part of a longitudinal study into orthographic learning (van Viersen et al., 2021). Ethical approval was provided by the Ethics Committee of the University of Amsterdam (case no. 2017-CDE-8332). None of the children had sensory deficits or neurodevelopmental disorders. Children with dyslexia or a preferred language other than Dutch were excluded. Sample characteristics are provided in Table 2.

**Table 2:** Sample characteristics

Variable	Grade 3				Grade 5			
	*M*	*SD*	Min	Max	*M*	*SD*	Min	Max
Age (months)	106.59	5.14	97	124	128.98	5.94	109	149
Word reading^a^	11.73	3.00	5	19	10.07	3.79	1	19
Nonword reading^a^	11.18	3.33	1	19	10.08	3.63	1	19
Vocabulary^b^	105.18	10.87	74	128	106.26	10.37	77	142
Syntactic skills^a^	10.40	2.42	5	15	9.54	2.39	4	16

*M* Mean, *SD* standard deviation, *Min* minimum, *Max* maximum

^a^Standard score (*M* = 10, *SD* = 3). ^b^Standard score (*M* = 100, *SD* = 15). See Instruments for description of the tasks

### Instruments

#### Measures of within-word processes

Word-level subskills associated with within-word processing were measured with three discrete tasks: (1) discrete nonword reading, (2) discrete word reading, and (3) discrete digit naming.

For discrete words, items were 36 four-letter words selected from the CELEX database (Baayen et al., 1993) containing either vowel digraphs or consonant clusters (see van den Boer et al., 2016; van Viersen et al., 2022). For discrete nonwords, items were 36 four-letter nonwords created from the list of words used for the discrete word-reading task. This was done by interchanging onsets and rhymes or individual letters and matching them to the words on onset and consonant–vowel structure (e.g., words: boer, vuur, stil, werk; nonwords: bijn, veul, stes, werm; see van den Boer et al., 2016; van Viersen et al., 2022). For discrete digits, items were four different digits (i.e., 2, 3, 5, 6) that each appeared nine times throughout the task. The digits were selected to have monosyllabic number words to correspond to the items in the (non)word reading tasks and keep naming demands matched across tasks.

In each task, items were displayed one at a time in black 20-point Consolas on a white screen using DMDX (Forster & Forster, 2003). Children had to read or name each item aloud upon appearance, starting with four practice items. A key press from the experimenter controlled the progression to the next item. Responses were audio recorded to capture both onset latency and articulation duration. Vocal responses were subsequently processed in CheckVocal 2.3.1 and 3.0a (Protopapas, 2007). Waveform and spectrogram of vocal responses were displayed to mark speech offsets and determine the total naming/reading time per item or array (i.e., response time [RT], including onset latency and articulation duration). Errors were marked within the program. For the discrete tasks, RTs were converted to reading rates (i.e., number of items per second) and averaged for each participant across correct items. The raw score was thus the mean reading/naming time across correct items (see e.g., Altani et al., 2018). For the serial tasks, the number of correctly read/named items in the array was divided by the total reading/naming time (i.e., including correct and incorrect items). This approach aligns with oral reading fluency measures penalizing decoding errors (see also van Viersen et al., 2022). Cronbach’s α on the current sample (Grade 3/Grade 5) was .94/.96 for discrete nonwords, .96/.97 for discrete words, and .96/.97 for discrete digits.

#### Sequential processing efficiency

Sequential processing efficiency was measured with a serial digit-naming task (e.g., Altani et al., 2020; de Jong, 2011; Protopapas et al., 2018). Thirty-six digits (i.e., nine repetitions of digits 2, 3, 5, and 6) were displayed all at once in four rows of nine items using DMDX. Children were asked to name the full array of digits upon appearance from top left to bottom right as fast and accurately as possible, starting with four practice items. For the serial tasks, vocal responses were also processed in CheckVocal 2.3.1 and 3.0a (Protopapas, 2007). Reading rates were computed, including both correct and incorrect responses (i.e., 36 divided by the total RT). The raw score was the total naming time for the entire array. This approach aligns with common practice in scoring serial naming tasks (see also e.g., Altani et al., 2018; van den Boer et al., 2016; van Viersen et al., 2022). Reliabilities of paper and pencil versions of digit naming tasks lie between .79 and .87 in this age group (Evers et al., 2009–2012).

#### Vocabulary

Receptive vocabulary was measured with the *Peabody Picture Vocabulary Test NL* (PPVT-NL; Schlichting, 2005). Children had to select the corresponding picture out of four alternatives matching a verbally presented target word. The test consists of 17 sets of 12 words and starts with the entry set consistent with the child’s age. The start set, from which correct answers start to count, is the first set in which the child correctly identifies at least four pictures. The end set, after which the test is terminated, is the last set in which the child fails to identify nine or more pictures. The raw score is the number of correctly identified pictures in the administered sets, including the non-administered sets that preceded the start set (which are automatically scored as correct). Standard scores are available using age-based norms (*M* = 100, *SD* = 15). The reliability of the PPVT-NL is considered good (Egberink et al., 2017).

#### Syntactic skills

Syntactic skills were measured using the Formulated Sentences subtest of the *Clinical Evaluation of Language Fundamentals-4 NL* (CELF; Kort et al., 2010) tapping expressive grammar. Children had to create sentences about pictured situations using a verbally presented target word, for example, by using (1) the word ‘*eindelijk*’ (finally) to make a sentence about a picture showing a boy handing in his homework (simpler item), or (2) the words ‘*in plaats van*’ (instead) to describe a situation in which a boy chooses a book from a shelf (more difficult item). Quality of the produced sentences was evaluated following the guidelines in the manual (i.e., 1 or 2 points per sentence based on correctness and complexity). The raw score was the total number of scored points. Standard scores are based on age-grouped norms (*M* = 10, *SD* = 3). Internal consistency of the subtest is .78 (Evers et al., 2009–2012).

#### Measures of reading fluency

Reading fluency was assessed with a set of serial reading tasks of word lists, nonword lists, and a text, including variants with simple and more demanding items.

Two separate serial reading tasks were used to assess reading of simple word and nonword lists (de Jong, 2011; Protopapas et al., 2018; van den Boer et al., 2016). The simple word list contained 36 high-frequency four-letter words, and the simple nonword list contained 36 four-letter nonwords (see van den Boer et al., 2016; van Viersen et al., 2022). The items in these tasks are equally complex as those in the discrete tasks but are instead displayed in serial format. Items were matched to the discrete word and nonword reading tasks based on onset phoneme, length, consonant–vowel structure, and (for words) frequency. Items were displayed in four rows of nine items using DMDX. Children were asked to read the (non)words aloud from top left to bottom right as fast and accurately as possible, starting with four practice items. Raw scores are the total reading times for the full arrays of (non)words (as in, e.g., Altani et al., 2018).

Reading fluency of complex word and nonword lists was measured using the Dutch *Eén Minuut Test* (EMT; Brus & Voeten, 1999) and *Klepel* (van den Bos, lutje Spelberg, Scheepstra, & de Vries, 1994). Both tests comprise a list of 116 items of increasing difficulty (i.e., one to four syllables), making the items more difficult than the four-letter (non)words in the simpler lists. Children had to read as many (non)words as possible within one (words) and two (nonwords) minutes, respectively. Raw scores are the number of correctly read (non)words within the time limit. Standard scores are based on grade-level norms per semester (*M* = 10, *SD* = 3). Test–retest reliability is .90 for EMT and .92 for Klepel (Evers et al., 2009–2012).

Text-reading fluency was measured using an adaptation of the Dutch text *Knuffelapen* (Cuddly Monkeys; L. Bazen, personal communication, September 2018). The text contained 246 words and covered one page of connected text. The task was a mix of narrative and expository text. Sentence length and difficulty were aligned with the level generally encountered in textbooks in mid-primary education. This text was originally a silent reading task and contained small tasks to monitor progress (e.g., “now clap your hands”). For the present study, the small tasks for progress-monitoring were skipped, and instead children were asked to read the text out aloud as fast and accurately as possible. Reading errors were scored and reading time was recorded. The score was the number of correctly read words per second.

#### Procedure

Children were tested during one individual session in February or March 2019. Testing took place in a quiet room at school and was conducted by trained and supervised (under)graduate students. The administered tasks were part of a larger test battery, for which assessment took between 40 and 60 min. Ample breaks were provided throughout testing.

## Results

### Data screening

Nine univariate outliers (Grade 3: 5 across 5 different variables; Grade 5: 4 across 4 different variables) were adjusted through winsorization using percentiles to decrease their influence. Missing data ranged from 0.0 to 2.6% across variables in Grade 3 and 0.0–3.3% in Grade 5. All variables were approximately normally distributed (see Table 3). The scales of the vocabulary and syntactic skill measures were adjusted to align with the other predictor variables. Fluency outcomes were all reported as items (words or digits) per second. Data and R code are publicly available through the OSF at https://osf.io/mkurw/.

**Table 3:** Descriptives for predictors and outcomes

Variables	Grade 3						
	*n*	*M*	*SD*	Min	Max	Skew	Kurt
Discrete nonwords^a^	77	0.80	0.12	0.55	1.08	0.37	− 0.50
Discrete words^a^	76	0.96	0.13	0.70	1.27	0.33	− 0.22
Discrete digits^a^	77	1.02	0.13	0.74	1.34	0.22	− 0.09
Serial digits^b^	75	1.66	0.31	1.08	2.34	0.05	− 0.56
Vocabulary^c^	77	1.13	0.09	0.91	1.33	− 0.23	− 0.37
Syntactic skills^d^	77	2.55	0.44	1.50	3.40	− 0.20	− 0.71
Simple nonword list^b^	76	1.07	0.30	0.41	1.83	0.21	− 0.69
Complex nonword list^e^	77	0.80	0.28	0.12	1.42	0.10	− 0.33
Simple word list^b^	75	1.72	0.34	0.81	2.51	0.02	0.01
Complex word list^e^	77	1.02	0.22	0.55	1.52	0.05	− 0.22
Text^e^	77	2.03	0.52	1.01	3.38	0.54	0.25
	Grade 5						
Discrete nonwords^a^	60	0.85	0.13	0.61	1.12	0.20	− 0.73
Discrete words^a^	60	0.99	0.12	0.75	1.31	0.54	0.07
Discrete digits^a^	60	1.07	0.12	0.81	1.37	0.35	− 0.04
Serial digits^b^	59	1.93	0.34	1.31	2.68	0.15	− 0.45
Vocabulary^c^	61	1.28	0.09	1.07	1.49	0.30	− 0.16
Syntactic skills^d^	61	2.84	0.39	2.00	3.80	0.22	− 0.58
Simple nonword list^b^	60	1.20	0.37	0.42	1.98	− 0.03	− 0.70
Complex nonword list^e^	61	0.96	0.32	0.27	1.75	0.03	− 0.48
Simple word list^b^	60	1.90	0.38	1.00	2.71	− 0.03	− 0.39
Complex word list^e^	60	1.19	0.25	0.64	1.75	0.05	− 0.54
Text^e^	61	2.46	0.56	1.24	3.84	0.16	− 0.38

*M* mean, *SD* standard deviation, *Min* minimum, *Max* maximum, *Skew* skewness, *Kurt* kurtosis, *Vocabulary* receptive vocabulary, *Syntactic skills* expressive grammar

^a^Mean naming/reading time in seconds across correct items. ^b^Rate (items per second). ^c^Raw score rescaled by dividing by 100. ^d^Raw score rescaled by dividing by 10. ^e^Fluency (correct items per second)

The correlations among word- and text-level predictors per grade are displayed in Table A.1 (see Supplementary Information). Correlations among the discrete tasks were high. This is in line with previous studies including multiple discrete tasks to measure subskills associated with within-word processing (Maassen & Bakker, 2001; see e.g., Logan et al., 2011; Protopapas et al., 2013a; Zoccolotti et al., 2014). Notably, the relation between discrete nonwords and discrete digits was higher in Grade 5 than in Grade 3. Relations of discrete tasks with serial digits were small to moderate in both grades. Vocabulary and syntactic skills were moderately related to each other, but not to the other predictors in both grades.

Correlations between the predictors and reading fluency outcomes for each grade are displayed in Table 4. The association between discrete nonword reading and all fluency measures remained significant and rather stable across grades. In contrast, the correlation between discrete word reading and lists of words and nonwords dropped from Grade 3 to Grade 5. What stood out was the increasing relation between serial digit naming and all fluency outcomes—including nonword lists—from small-to-medium correlations in Grade 3 (i.e., ranging from .30 to .43) to medium-to-large correlations in Grade 5 (ranging from .45 to .62). This also held for text-reading fluency, for which the correlation with serial digit naming changed from low and non-significant in Grade 3 (*r* = .22) to substantial and significant (*r* = .54) in Grade 5. Additionally, vocabulary was significantly associated only with the more demanding fluency measures, including the complex word list and text reading in Grade 5; yet, it showed no significant correlations in Grade 3. Finally, syntactic skills showed a small but significant correlation with text-reading fluency, again only in Grade 5.

**Table 4:** Pearson’s correlations between predictor variables and reading fluency outcomes for Grades 3 and 5

Predictor variables	Nonwords^a^		Words^b^		Text^e^
	Simple	Complex	Simple	Complex	
*Grade 3*					
Discrete nonwords	.65***	.62***	.58***	.56***	.47***
Discrete words	.45***	.46***	.41***	.42***	.30**
Discrete digits	.26*	.22	.22	.18	.10
Serial digits	.43***	.37**	.43***	.30**	.22
Vocabulary	− .09	− .04	.01	.14	.11
Syntactic skills	− .03	.12	− .01	.24*	.12
*Grade 5*					
Discrete nonwords	.59***	.65***	.47***	.64***	.58***
Discrete words	.27*	.36**	.28*	.33*	.28*
Discrete digits	.16	.25	.19	.23	.20
Serial digits	.45***	.50***	.62***	.53***	.54***
Vocabulary	.18	.21	.24	.27*	.28*
Syntactic skills	.14	.09	.17	.22	.26*

*p < .05. **p < .01. ***p < .001

### Hierarchical regressions

Contributions of subskills associated with word- and text-level processing to fluent reading of lists of (non)words and connected text were assessed with hierarchical regressions per fluency outcome in *base R* version 4.1.2 (R Core Team, 2022). This approach allowed us to assess the unique effect of sequential processing efficiency and vocabulary/syntactic skills, after subskills associated with within-word level processing were controlled. Accordingly, the discrete tasks were entered first. Subsequently, serial digit naming, vocabulary, and syntactic skills were added in three distinct steps. The analyses were conducted separately for Grades 3 and 5. Standardized coefficients (β) and *R*^2^_change_ are reported and evaluated in Table 5 and 6, respectively.

**Table 5:** *R*^2^ changes for word- and text-level predictors of fluency outcomes per grade

		Grade 3					Grade 5				
		Nonwords		Words		Text	Nonwords		Words		Text
Step	Predictors	Simple	Complex	Simple	Complex		Simple	Complex	Simple	Complex	
1	Discrete tasks	.42***	.51***	.37***	.44***	.35***	.43***	.36***	.17**	.42***	.39***
2	Serial digits	.07**	.03*	.08**	.06**	.04*	.05	.13*	.29***	.13**	.19**
3	Vocabulary	.00	.00	.02	.05**	.07**	.00	.03	.00	.03	.04
4	Syntactic skills	.00	.00	.00	.01	.00	.07	.00	.00	.01	.02
	Total *R*^2^	.50	.55	.47	.56	.46	.55	.52	.46	.60	.64

Hierarchical regressions with four steps. Asterisks indicate significance of *R*^2^_change_

*S* simple list, *C* complex list

*p < .05. ** *p* < .01. ***p < .001

**Table 6:** Standardized coefficients for word- and text-level predictors of fluency outcomes per grade

	Grade 3					Grade 5				
	Nonwords		Words		Text	Nonwords		Words		Text
Predictors	Simple	Complex	Simple	Complex		Simple	Complex	Simple	Complex	
Discrete nonwords	.70***	.71***	.56**	.66***	.69***	.99***	.79**	.52*	.93***	.91***
Discrete words	− .07	.05	− .02	− .10	− .24	− .30	− .00	.13	− .15	− .17
Discrete digits	− .31*	− .28*	− .20	− .19	− .14	− .19	− .49	− .61	− .48	− .49
Serial digits	.32**	.23*	.32**	.27**	.21*	.08	.39*	.56**	.33*	.40*
Vocabulary	− .01	− .09	.17	.15	.27*	− .05	.17	− .03	.14	.15
Syntactic skills	− .06	.09	− .07	.13	− .01	.36	.03	.05	.16	.20

Standardized regression coefficients of the final model with all predictors included

*p < .05. **p < .01. *** *p* < .001

In Grade 3, serial digit naming contributed a significant but small amount of additional variance to all reading fluency outcomes after discrete tasks were taken into account. Moreover, vocabulary contributed a significant but small amount of additional variance to reading fluency of complex word lists and texts after word level processes were considered. In Grade 5, serial digit naming also contributed a significant amount of variance to all reading outcomes, except for fluent reading of simple nonword lists, after discrete tasks were taken into account. The amount of variance that serial digit naming contributed in Grade 5 is medium to large, which is considerably larger than the small amount contributed in Grade 3. There were no additional significant contributions of vocabulary or syntactic skills in Grade 5. The amount of total explained variance by the predictors was largely comparable between grades, except for text-reading fluency, for which the amount of explained variance was larger in Grade 5 than in Grade 3. However, as follows from the findings above, the relevant contributions of the predictors reflecting subskills associated with within- or between-word processing differed between grades; there was a larger contribution of discrete tasks in Grade 3 and larger contribution of serial digit naming (i.e., sequential processing efficiency) in Grade 5.

Table 6 shows the standardized regression coefficients of the final model including all predictors. As a result of the high correlations between the discrete tasks, a suppression effect occurred. A suppression effect is suspected when the sign of the regression coefficient of an independent variable is opposite to what is expected based on its correlation with the outcome variable (Tabachnick & Fidell, 2007). Moreover, the variable only functions as a suppressor for those variables whose regression coefficients increase when this variable is added to the model (Conger, 1974). Here, both discrete word reading and discrete digit naming had sizable negative but nonsignificant regression coefficients, whereas correlations between these variables and fluency outcomes were positive. Entering the discrete tasks and serial digit naming (due to its overlap with discrete digit naming) into the model in different orders (see Table A.2 in the Supplementary Information) revealed that both discrete words and discrete digits acted as suppressors. However, when both were included in the model, and serial digit naming was also added, discrete digit naming remained as the main suppressor. Inclusion of discrete digit naming led to increased effects of both discrete nonwords and serial digit naming, and this effect was stronger in Grade 5 than in Grade 3. This indicates that the effects of discrete nonwords and serial naming on the fluency outcomes are unrelated to what is shared with discrete digit naming.

Given the suppression effect, coefficients for the discrete predictors were not interpreted. In general, the results in Table 6 confirmed that, after within-word processes were controlled, serial digit naming had a significant medium effect on all fluency outcomes in Grade 3 and a medium-to-large effect on fluency outcomes in Grade 5 (except for simple nonword lists). There was also a significant effect of vocabulary on text-reading fluency in Grade 3, but not on the complex word list. There were no significant effects of vocabulary or syntactic skills on fluency outcomes in Grade 5. The significant contribution of vocabulary to the more complex fluency outcomes in Grade 3, despite nonsignificant zero-order correlations, was not the result of a suppression effect. Rather, controlling for the other variables in the model allowed for a significant effect to emerge by reducing the error variance.

### Commonality analyses

Commonality analyses conducted in R with *yhat* (Nimon et al., 2008) revealed the unique contribution of serial digit naming and vocabulary/syntactic skills to the various fluency tasks. These analyses also helped to dissect the suppression effect, as the outcomes can illustrate unique effects among tasks that are highly correlated (Nimon & Reio, 2011). Table A.3 (see Supplementary Information) shows the unique and total contribution of the discrete tasks to the reading fluency outcomes in each grade. According to Capraro and Capraro (2001), suppressors can also be identified by low variance contributions, in addition to large but often nonsignificant standardized regression coefficients. The suppressive effect of discrete digit naming was thus further confirmed by its low variance contribution to the fluency outcomes compared to its regression coefficients displayed in Table 6. In contrast, discrete nonwords contributed considerable unique and shared variance to all reading outcomes in both grades, in line with the strong betas.

Table 7 displays the unique and total contributions of serial digit naming, vocabulary, and syntactic skills. As expected, serial digit naming contributed most unique and total variance after discrete tasks were considered, and these proportions were larger in Grade 5 than in Grade 3. This pattern corresponds to the previously found developmental shift in dominant processes underlying fluency. However, the concurrent decreasing influence of within-word processes with increasing reading experience was not evident from all discrete tasks (see Table A.3 in the Supplementary Information): the total contribution of discrete word reading decreased over time, whereas the contribution of discrete nonwords did not. Furthermore, vocabulary added unique variance to complex word lists and texts in Grade 3, which was an amount comparable to serial digit naming. In Grade 5, there was a small unique contribution of syntactic skills to text reading. This did not surface in the hierarchical regressions.

**Table 7:** Variance proportions of sequential processing efficiency, vocabulary, and syntactic skills for fluency outcomes per grade (after controlling for discrete tasks)

	Grade 3		Grade 5	
	Unique	Total	Unique	Total
*Simple nonword list*				
Serial digits	.03	.18	.06	.21
Vocabulary	.00	.01	.00	.01
Syntactic skills	.01	.00	.01	.01
*Complex nonword list*				
Serial digits	.03	.14	.08	.25
Vocabulary	.00	.00	.00	.02
Syntactic skills	.01	.02	.00	.00
*Simple word list*				
Serial digits	.05	.19	.25	.39
Vocabulary	.02	.00	.02	.04
Syntactic skills	.01	.00	.00	.02
*Complex word list*				
Serial digits	.02	.09	.09	.28
Vocabulary	.03	.03	.01	.03
Syntactic skills	.02	.06	.01	.03
*Text reading*				
Serial digits	.01	.05	.11	.29
Vocabulary	.02	.01	.00	.04
Syntactic skills	.00	.01	.04	.04

## Discussion

In this study, we aimed to gain insight into the contribution of between-word processing (as indicated by sequential processing efficiency) and subskills related to text-level processing (vocabulary and syntactic skills) to a wide range of reading fluency tasks. We specifically investigated (a) sequential processing efficiency at various points during development (in Grades 3 and 5), (b) its contribution to reading fluency of simple and complex lists of words and nonwords and a more complex text, and (c) how subskills typically associated with word-level reading and text-level comprehension processes may uniquely contribute to these reading fluency outcomes. Our hypothesis on the contribution of sequential processing efficiency to reading fluency outcomes, beyond within-word processes, was largely confirmed. However, findings were more mixed regarding the contribution of subskills related to text-level processing to fluent reading of complex word lists and a more complex text.

### Between-word processing in reading fluency

As expected, sequential processing efficiency, defined as the ability to coordinate the processing of multiple words at the same time and indexed by a serial digit RAN task, contributed uniquely to almost all reading fluency measures across intermediate and more advanced readers. The only exception was simple nonwords in Grade 5, to which sequential processing efficiency did not contribute additionally after discrete nonword reading was controlled. In addition, the contribution of sequential processing efficiency increased with grade for all fluency measures, after controlling for within-word processes. Previous studies have claimed that a critical aspect of reading fluency and serial digit naming—which is missing from individual item naming or single-word reading (discrete digit-naming and discrete word-reading tasks)—is the requirement of self-paced or endogenously controlled processing of multiple items (see Altani et al., 2020; Gordon & Hoedemaker, 2016; Protopapas et al., 2018; Zoccolotti et al., 2014). In other words, the reader is required to efficiently schedule the processing of multiple items in these tasks, including regulation of the pace and the extent of overlap of processing stages between words (see Protopapas et al., 2018). Our findings confirm that efficient between-word processing becomes more important as children become more skilled readers and word identification less demanding (see also Altani et al., 2018, 2020; Protopapas et al., 2013a).

The intermediate reading level of the Grade 3 children seems to reflect a transitional phase in reading fluency development, where subskills related to within-word processing are still a major factor, but sequential processing efficiency begins to play a role in fluency when tasks contain simple, short words or nonwords that are easy to identify. However, as it might still be more demanding to identify longer words and nonwords, this may limit the ability to partially process adjacent words and thus limit efficient between-word processing in complex tasks. Therefore, sequential processing efficiency does not yet contribute to fluency performance as much in intermediate-level readers as later in reading development for the more advanced readers in Grade 5.

Together, the findings above extend the evidence for the essential role of sequential processing efficiency for fluent reading of simple word lists and texts (e.g., Altani et al., 2020; de Jong, 2011; Protopapas et al., 2013a; van den Boer & de Jong, 2015) to complex word lists and texts as well as simple and complex lists of nonwords. Previous evidence (Altani et al., 2020; Georgiou et al., 2022; Protopapas et al., 2018; Zoccolotti et al., 2014) on simple word lists and simple texts thus cannot be attributed to a methodological artifact, that is, that both serial digit naming and serial word reading involve scanning easy items that pose no considerable item-level processing difficulties. Therefore, our study corroborates the idea that sequential processing efficiency at the between-word level may be a separate component of reading fluency, and its further development may be necessary for building fluency.

#### The suppression effect

We confirmed a previously well-documented suppression effect from discrete word naming and—especially—discrete digit naming to discrete nonwords and serial digits in the prediction of fluency outcomes (e.g., Altani et al., 2018; Logan et al., 2011; Zoccolotti et al., 2014). Although counterintuitive, this observed suppression effect further supports the idea that individual differences in word recognition (discrete words) and lexical access (discrete digits) are relatively less important for reading fluency. Generally, an independent variable that acts as a suppressor removes variance in other independent variables that is irrelevant for the relation with a dependent variable. In other words, the suppressor “purifies” one or more independent variables by removing their shared-but-unrelated-to-the-dependent-variable variance, and thereby, increases their predictive power. Technically, a suppression effect is indicated by the suppressor contributing little to no variance to the dependent variable, but at the same time having a substantial beta weight, most likely in the opposite direction compared to what would be expected from the correlations (Capraro & Capraro, 2001; Tabachnick & Fidell, 2007). Our results clearly indicated that both discrete words and discrete digits acted as suppressors. Further analyses showed that discrete digit naming was the main suppressor, substantially raising the regression coefficients of both discrete nonwords and serial digit naming.

The remaining question concerns what is being suppressed by discrete digit naming. What does discrete digit naming capture in terms of *shared but irrelevant* variance in discrete nonwords and serial digit naming that leads to an increase in their relationship with reading fluency? Discrete digit naming shares variance with serial digit naming and discrete nonword reading as it requires common processes related to successful print-to-sound mapping of individual items. Therefore, the observed suppressive effect of discrete digits on discrete nonwords suggests that shared processes of individual item identification speed or rapid access and retrieval of phonological code is not the reason that discrete nonword reading predicts reading fluency. Instead, the unique variance captured by discrete nonword reading in reading fluency presumably reflects efficient processing specific to (within-word) multi-element graphophonemic mapping (see Zoccolotti et al., 2014, for a similar argument). The observed effect of discrete naming on serial naming has been proposed to suppress shared variance related to individual item naming speed and lexical retrieval, allowing serial naming to capture processes specific to the sequential nature of serial naming and reading-fluency tasks, regarding efficient processing of successive items (cf. Altani et al., 2018; Logan & Schatschneider, 2014; Logan et al., 2011; Protopapas et al., 2013a).

### Text-level subskills in reading fluency

Concerning the hypothesis that vocabulary would contribute uniquely to fluent reading of complex word lists, our expectations were two-fold. As evidence for the influence of vocabulary seems weaker in more transparent orthographies than in opaque orthographies (English, French; Taylor et al., 2015; Krepel et al., 2021; Nation & Snowling, 2004; Ouellette, 2006; Ricketts et al., 2007, 2016; Dutch; e.g., de Jong, 2011; de Jong & van der Leij, 2002), we expected that in intermediate-level readers, for whom word identification of longer/less frequent words is still demanding, vocabulary would contribute to fluent reading of complex word lists after subskills related to word-level processing are controlled. However, overall effects of vocabulary on reading fluency turned out to be negligible in Grade 3 readers. In contrast, for more advanced readers we assumed that word recognition is automated to such an extent that vocabulary knowledge has no role. Indeed, as expected, we found no effects of vocabulary on reading fluency in the more advanced readers. Our findings thus further support the suggestion that vocabulary seems less prominently involved in processing mechanisms for fluent word reading in more transparent orthographies, adding that this appears to hold across reading levels. Another interpretation is that semantic requirements at the individual word recognition level were already captured by measures of discrete words, leaving no additional variance to be captured by vocabulary.

Regarding the fluent reading of connected text, we hypothesized that vocabulary would contribute to text reading fluency across reading skill levels. Indeed, there was a small but significant effect of vocabulary on text-reading fluency in intermediate-level readers after accounting for word-level processes, but not in more advanced readers. This is in alignment with previous findings for sentence-reading fluency on the same sample of intermediate-level readers (van Viersen et al., 2022). Yet, the effect is smaller for text-reading fluency than for sentence-reading fluency. It is possible that the two measures slightly differ in word processing demands, as word length and frequency differed between the (previous) sentence- and (current) text-reading tasks. In general, the small effects suggest that vocabulary may not come into play before sentence/text reading is complex or challenging enough so that vocabulary becomes necessary for efficient word recognition and semantic integration.

We also hypothesized that syntactic skills would contribute to text-reading fluency in the more advanced readers. Despite there being an association, this relation disappeared after controlling for word-level processes and vocabulary. However, syntactic skills did contribute some unique variance to text-reading fluency in the more advanced readers in the commonality analyses. This may point towards (the start of) a developmental shift in older children, for whom word recognition and coordination of between-word processing may be automated to such an extent that they have enough resources available to allow for text processing. In contrast, intermediate-level readers may still spend too much time on word identification and/or are still developing their sequential processing efficiency. This more laborious processing may lead to the dedication of more resources to word-level processing, leaving fewer resources available for the processing of supra-lexical elements.

Alternatively, the lack of separate effects of vocabulary and syntactic skills might be explained by studies showing that language ability represents a unitary construct in young children (Bates & Goodman, 1997; Klem et al., 2015; Language & Reading Research Consortium, 2015; Tomblin & Zhang, 2006). Grammar development is highly driven by vocabulary size (see e.g., Bates & Goodman, 1997) and only starts to emerge as a separable language skill from Grade 3 on (Language & Reading Research Consortium, 2015). It could be that in our measures of text- and sentence-reading fluency, which do not contain clear manipulations in vocabulary or syntactic difficulty, language effects may be driven by the skill that is the most reliable measure from a psychometric viewpoint (i.e., here vocabulary) even up to Grade 5 (see also e.g., Lervåg et al., 2018; Protopapas et al., 2013b).

### Implications

Our findings support the conceptual framework of Hudson et al. (2009) regarding fluency as a complex multifaceted construct that combines multiple and interacting lower- and higher-level processes (see also Berninger et al., 2010; Katzir et al., 2006). Yet, although multiple word-level processes and their interactions are represented in the model, a clear conceptualization of how children move from processing single words to multiple words and sentences is missing. This is hardly a criticism of the model itself but illustrates current gaps in knowledge of the underlying mechanisms of reading fluency development.

The shift from reading individual words to multiple words and sentences seems to require additional processes at the between-word level. Rather than simply putting words next to each other, it seems that the reader needs to actively process word sequences in a cascading (i.e., partially overlapping) manner instead. Interestingly, RAN (measured by serial digit naming) is represented in the model. However, serial naming is still commonly viewed as reflecting sublexical (‘decoding’) fluency, both in empirical research and theoretical frameworks (e.g., Hudson et al., 2009; Kim & Wagner, 2015). Yet, our findings suggest that the relation between serial naming and reading fluency mainly reflects shared requirements of sequential processing efficiency at the between-word level—beyond decoding fluency captured by discrete nonword reading—that is critical for reading fluency and its development. As such, we contend that sequential processing efficiency (measured by serial digit naming) can be considered a subskill that adds an important piece of the puzzle at the between-word level, requiring a repositioning of the RAN concept in the model of Hudson et al. (2009).

Further evidence for the importance of between-word processing skill for reading fluency comes from studies on individuals with dyslexia suggesting, for example, a distinct and specific sequential processing impairment (e.g., Jones et al., 2009), as well as disproportionate difficulties with processing digits and words presented simultaneously in a multi-item version (i.e., matrix, list, sentences; e.g., Zoccolotti et al., 2013, 2015). Although, it is still unresolved where this sequential processing impairment stems from, it is consistent with the idea that reading words in lists or text reflects additional and distinct component processes (see Altani et al., 2017b; Ziaka & Protopapas, 2022; Ziaka et al., 2022).

From a developmental perspective, our results suggest that within-word level processing skills need to be in place by Grade 3, so that efficient between-word processing can also begin to develop to foster reading fluency. Sequential processing efficiency seems to be fully emerged by Grade 5, facilitating fluent reading of connected text. Moreover, word-level processes, both within and between words, must be at an adequate level to enable comprehension. Otherwise, children will not be ready to make the expected transition from learning-to-read to reading-to-learn and will risk falling behind in academic areas that become important in the higher grades of primary education (e.g., history, geography, etc.).

## Conclusion

This study confirms the importance of sequential processing efficiency for fluent reading of simple word lists and texts, while controlling for subskills associated with within-word processing, and extends these findings to complex word lists and texts, and simple and complex lists of nonwords. The findings hold across reading-skill levels. As expected, the contribution of sequential processing efficiency increased with reading-skill level, which is consistent with the idea that while subskills associated with within-word processing form the basis of fluent reading, further fluency development is driven by more efficient between-word processing. In contrast, general absence of effects of vocabulary on fluency measures supports the suggestion that vocabulary may be less prominently involved in processing mechanisms for fluent word identification in transparent orthographies.

## Supplementary Information

Below is the link to the electronic supplementary material.

Supplementary file1 (DOCX 34 kb)

## Footnotes

1 Discrete word reading and discrete digit naming are thus considered to cover within-word processes related to individual word recognition and/or direct lexical access.
