# Supplementary material for: Between-word processing and text-level skills contributing to fluent reading of (non)word lists and text
Source: Read Writ. 2024 Apr 3;38(3):671–97. doi: 10.1007/s11145-024-10533-8 (PMC11914343; doi:10.1007/s11145-024-10533-8)
Supplement: Supplementary file 1 — Supplementary file1 (DOCX 34 kb) [file 11145_2024_10533_MOESM1_ESM.docx]

**Table A.1**

*Pearson’s Correlations among Predictor Variables for Grades 3 (above diagonal) and Grade 5 (below diagonal)*

| Variables | 1. | 2. | 3. | 4. | 5. | 6. |
| --- | --- | --- | --- | --- | --- | --- |
| 1. Discrete nonwords^a^ | - | .81^***^ | .56^***^ | .40^***^ | −.16 | .05 |
| 2. Discrete words^a^ | .79^***^ | - | .76^***^ | .24^*^ | −.16 | .03 |
| 3. Discrete naming^a^ | .72^***^ | .81^***^ | - | .28^*^ | −.25^*^ | −.08 |
| 4. Serial naming^b^ | .39^**^ | .23 | .34^**^ | - | −.22 | −.14 |
| 5. Vocabulary^c^ | .16 | .13 | .09 | .02 | - | .42^***^ |
| 6. Syntactic skills^d^ | .03 | .13 | .00 | .03 | .40^**^ | - |

*Note*. See Table 2 for variable units.

^*^ *p* < .05. ^**^ *p* < .01. ^***^ *p* < .001.

**Table A.2**

*Standardized Coefficients for Word- and Text-Level Predictors of Fluency Outcomes per Grade*

|  | Grade 3 | | | | | |  | Grade 5 | | | | | |
| --- | --- | --- | --- | --- | --- | --- | --- | --- | --- | --- | --- | --- | --- |
|  | Nonwords | |  | Words | | Text |  | Nonwords | |  | Words | | Text |
| Predictors | S | C |  | S | C |  |  | S | C |  | S | C |  |
| *Model 1* |  |  |  |  |  |  |  |  |  |  |  |  |  |
| 1. Discrete nonwords | .59 | .68 |  | .58 | .63 | .54 |  | .57 | .53 |  | .31 | .53 | .49 |
| *Model 2* |  |  |  |  |  |  |  |  |  |  |  |  |  |
| 1. Discrete nonwords | .44 | .59 |  | .43 | .50 | .42 |  | .54 | .47 |  | .21 | .47 | .42 |
| 2. Serial digits | .31 | .20 |  | .33 | .29 | .27 |  | .13 | .29 |  | .45 | .27 | .34 |
| *Model 3a* |  |  |  |  |  |  |  |  |  |  |  |  |  |
| 1. Discrete nonwords | .73 | .77 |  | .57 | .71 | .70 |  | .79 | .68 |  | .40 | .77 | .73 |
| 2. Serial digits | .26 | .17 |  | .31 | .26 | .22 |  | .13 | .28 |  | .45 | .27 | .33 |
| 3. Discrete words | −.34 | −.21 |  | −.17 | −.24 | −.33 |  | −.33 | −.28 |  | −.24 | −.39 | −.41 |
| *Model 3b* |  |  |  |  |  |  |  |  |  |  |  |  |  |
| 1. Discrete nonwords | .65 | .75 |  | .55 | .64 | .57 |  | .87 | .80 |  | .57 | .88 | .85 |
| 2. Serial digits | .33 | .21 |  | .34 | .30 | .28 |  | .23 | .39 |  | .56 | .39 | .47 |
| 3. Discrete naming | −.34 | −.27 |  | −.21 | −.24 | −.25 |  | −.52 | −.51 |  | −.56 | −.64 | −.69 |
| *Model 4* |  |  |  |  |  |  |  |  |  |  |  |  |  |
| 1. Discrete nonwords | .69 | .73 |  | .54 | .68 | .68 |  | .88 | .78 |  | .51 | .88 | .84 |
| 2. Serial digits | .31 | .22 |  | .34 | .29 | .25 |  | .23 | .39 |  | .58 | .39 | .47 |
| 3. Discrete words | −.08 | .03 |  | .01 | −.06 | −.19 |  | −.02 | .06 |  | .16 | .01 | .02 |
| 4. Discrete digits | −.31 | −.28 |  | −.21 | −.21 | −.16 |  | −.51 | −.54 |  | −.65 | −.65 | −.70 |

*Note.* Standardized regression coefficients of the final model with all predictors included. S = simple list; C = complex list. 
^*^ *p* < .05. ^**^ *p* < .01. ^***^ *p* < .001.

**Table A.3**

*Variance Proportions of Discrete Tasks for Fluency Outcomes per Grade*

|  | Grade 3 | |  | Grade 5 | |
| --- | --- | --- | --- | --- | --- |
|  | Unique | Total |  | Unique | Total |
| Serial nonword reading |  |  |  |  |  |
| Discrete nonwords | .12 | .43 |  | .28 | .32 |
| Discrete words | .00 | .21 |  | .00 | .06 |
| Discrete digits | .02 | .07 |  | .08 | .02 |
| Nonword-list reading |  |  |  |  |  |
| Discrete nonwords | .09 | .39 |  | .24 | .39 |
| Discrete words | .00 | .22 |  | .00 | .11 |
| Discrete digits | .03 | .05 |  | .05 | .07 |
| Serial word reading |  |  |  |  |  |
| Discrete nonwords | .09 | .34 |  | .06 | .20 |
| Discrete words | .00 | .17 |  | .00 | .07 |
| Discrete digits | .01 | .05 |  | .05 | .04 |
| Word-list reading |  |  |  |  |  |
| Discrete nonwords | .09 | .32 |  | .26 | .38 |
| Discrete words | .00 | .17 |  | .00 | .09 |
| Discrete digits | .01 | .03 |  | .05 | .06 |
| Text reading |  |  |  |  |  |
| Discrete nonwords | .09 | .22 |  | .24 | .31 |
| Discrete words | .00 | .09 |  | .02 | .06 |
| Discrete digits | .02 | .01 |  | .03 | .04 |
